# Regeneration of skin appendages and nerves: current status and further challenges

**DOI:** 10.1186/s12967-020-02248-5

**Published:** 2020-02-03

**Authors:** Tingting Weng, Pan Wu, Wei Zhang, Yurong Zheng, Qiong Li, Ronghua Jin, Haojiao Chen, Chuangang You, Songxue Guo, Chunmao Han, Xingang Wang

**Affiliations:** 1 Department of Burns & Wound Care Center, Second Affiliated Hospital of Zhejiang University, College of Medicine, Hangzhou, 310009 China; 2 Department of Breast Surgery, Zhejiang Cancer Hospital, Hangzhou, 310022 China; 3 Department of Plastic Surgery, Second Affiliated Hospital of Zhejiang University, Hangzhou, 310009 China

**Keywords:** Skin appendages, Hair follicle, Neural regeneration, Tissue engineering, Regenerative medicine

## Abstract

Tissue-engineered skin (TES), as an analogue of native skin, is promising for wound repair and regeneration. However, a major drawback of TES products is a lack of skin appendages and nerves to enhance skin healing, structural integrity and skin vitality. Skin appendages and nerves are important constituents for fully functional skin. To date, many studies have yielded remarkable results in the field of skin appendages reconstruction and nerve regeneration. However, patients often complain about a loss of skin sensation and even cutaneous chronic pain. Restoration of pain, temperature, and touch perceptions should now be a major challenge to solve in order to improve patients’ quality of life. Current strategies to create skin appendages and sensory nerve regeneration are mainly based on different types of seeding cells, scaffold materials, bioactive factors and involved signaling pathways. This article provides a comprehensive overview of different strategies for, and advances in, skin appendages and sensory nerve regeneration, which is an important issue in the field of tissue engineering and regenerative medicine.

## Introduction

**Fig. 1:**
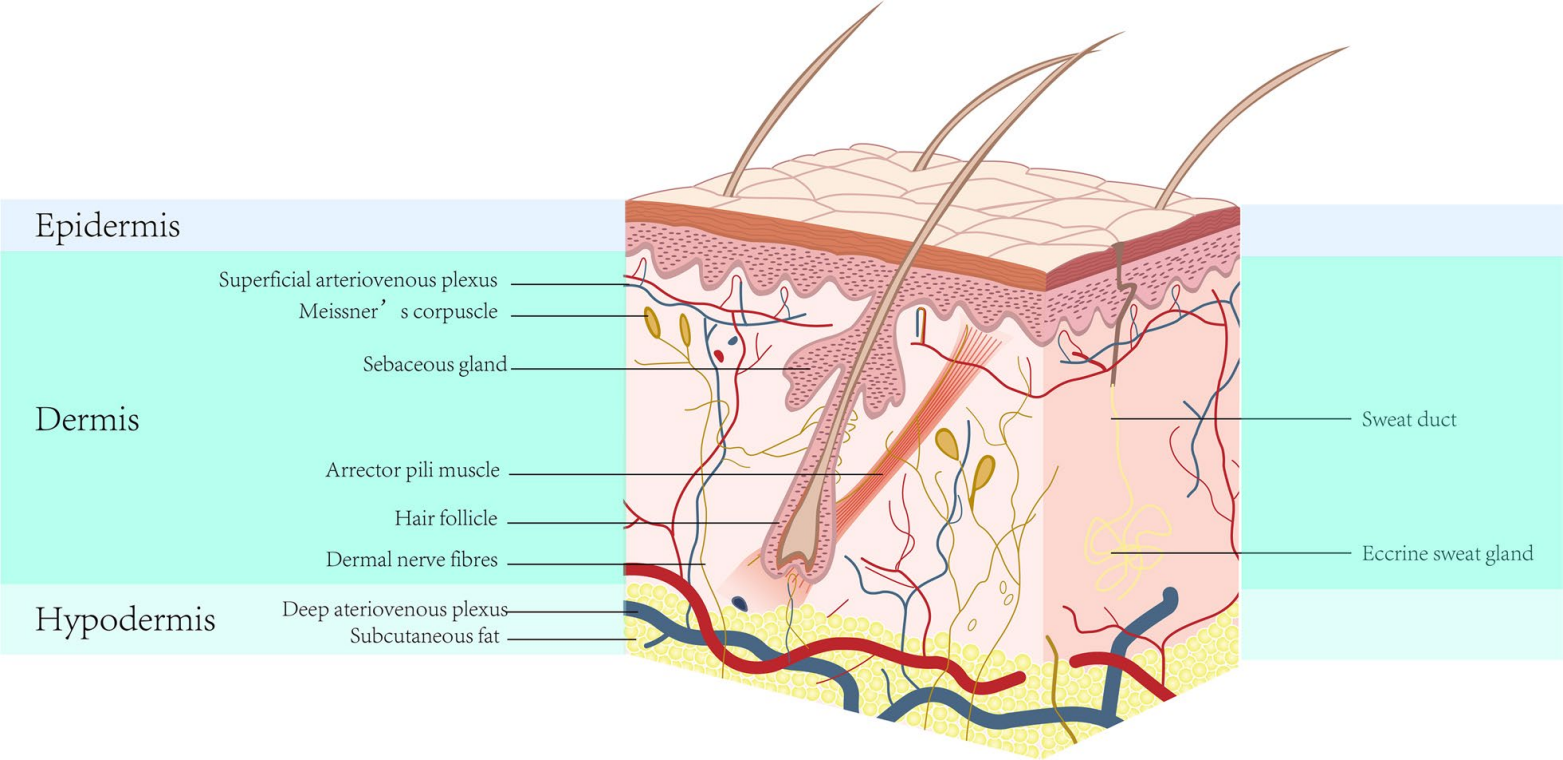
Schematic representation of the skin structure

The skin is composed of the epidermis, dermis and subcutaneous tissue, and is the largest organ of the human body (Fig. 1). The functions of the skin include (1) mechanical protection, (2) defence against the environment, (3) immune regulation, (4) body fluid regulation, (5) prevention of water loss from the body, (6) regulation of body temperature and (7) sensing of external stimuli. The skin is the outmost surface of the human body, and is thus susceptible to damage from the environment. Skin wounds caused by various acute and chronic factors, such as burns, surgery and diabetic ulcers, are very common, which imposes a heavy burden on society and the health of the general population [1, 2] (Table 1). Currently, the gold standard treatment for severe skin wounds is autologous skin grafting. However, the shortage of skin donor sites, secondary injury and risk of infection limit the application of autologous skin grafts. TES constitutes an alternative for wound coverage and tissue reconstruction.

**Table 1:** The structure of human skin and their functions

	Locations	Functions	References
Epidermis			
Melanocytes	Stratum basale	Production of melanosomes	[2]
Merkel cells	Stratum basale	Sensor	[2]
Keratinocytes	Stratum corneum Basement membrane	Secrete lipids, protease inhibitors, hydrolases, and antimicrobial peptides	[1, 2]
Langerhans cells	Stratum spinosum	Serving an immunologic role	[2]
Dermoepidermal junction	Epidermal and dermal layers	Regulates adhesion, movement, and growth of keratinocytes and fibroblasts4 Provides structural support for epidermis	[2]
Dermis			
Vasculature	Superficial plexus: papillary and reticular dermis Deep plexus: reticular dermis and hypodermis	Supply oxygen and nutrients to skin	
Arrector pili muscle	Attach to the hair follicle below the sebaceous glands	Pull the hair follicle	[2]
Arteries smooth muscle	Arterial wall	Perfusion blood	
Skeletal muscle	Face and anterolateral neck	Facilitate facial expression	
Nerves	Along arterioles and venules	Touch, pressure, temperature, itch, and pain sensation	[106]
Skin appendages			
Hair Follicle	Dermal layer of the skin	Form physical barrier, antibacterial, inhibit scar formation	[28]
Eccrine sweat glands	Palms and soles	Regulate body temperature	[61]
Apocrine sweat glands	Axillary and anogenital regions	Cause the characteristic smell of body	[61]
Sebaceous glands	Hair follicle and the arrector pili muscle	Secrete sebum and lubricate, protect against microorganisms	[78–80]
Hypodermis	Beneath the dermis and above the muscle	Insulate from the cold and violent trauma, provides buoyancy, storing energy	[2]

Tissue-engineered skin is a new type of tissue graft that can be used to directly treat wounds, induce cell migration, promote vascularisation and epithelisation, and accelerate wound healing [3]. TES is mainly composed of biomaterials, cells and bioactive factors. The ideal TES should include all of the skin appendages and layers (epidermis and dermis), and establish a functional vascular and nerve network, further promoting scar-free integration into the surrounding host tissue [4, 5]. Over the past several decades, there have been significant improvements in TES, with many engineered skin products being developed for clinical usage. Currently, TES mainly uses biological and synthetic materials combined with cells cultured in vitro to generate functional tissues, such as biomaterial dressings, cell-based skin substitutes, epithelial materials, dermal replacement materials and epithelial/dermal replacement materials [6]. However, these TES products can only form epidermal or dermal layers and fail to form effective skin appendages. Neurological recovery of long-term scarred skin remains a challenge [7]. All of these factors can seriously affect patient quality of life. Partial regeneration of skin function can pose heavy physical and emotional burdens on patients and impact their integration into society and daily lives. With the development of medical technology, there is a requirement for higher quality wound repair to achieve complete skin regeneration. Therefore, promoting the reconstruction and regeneration of skin appendages and nerves has become an important topic in the fields of tissue engineering and regenerative medicine.

**Fig. 2:**
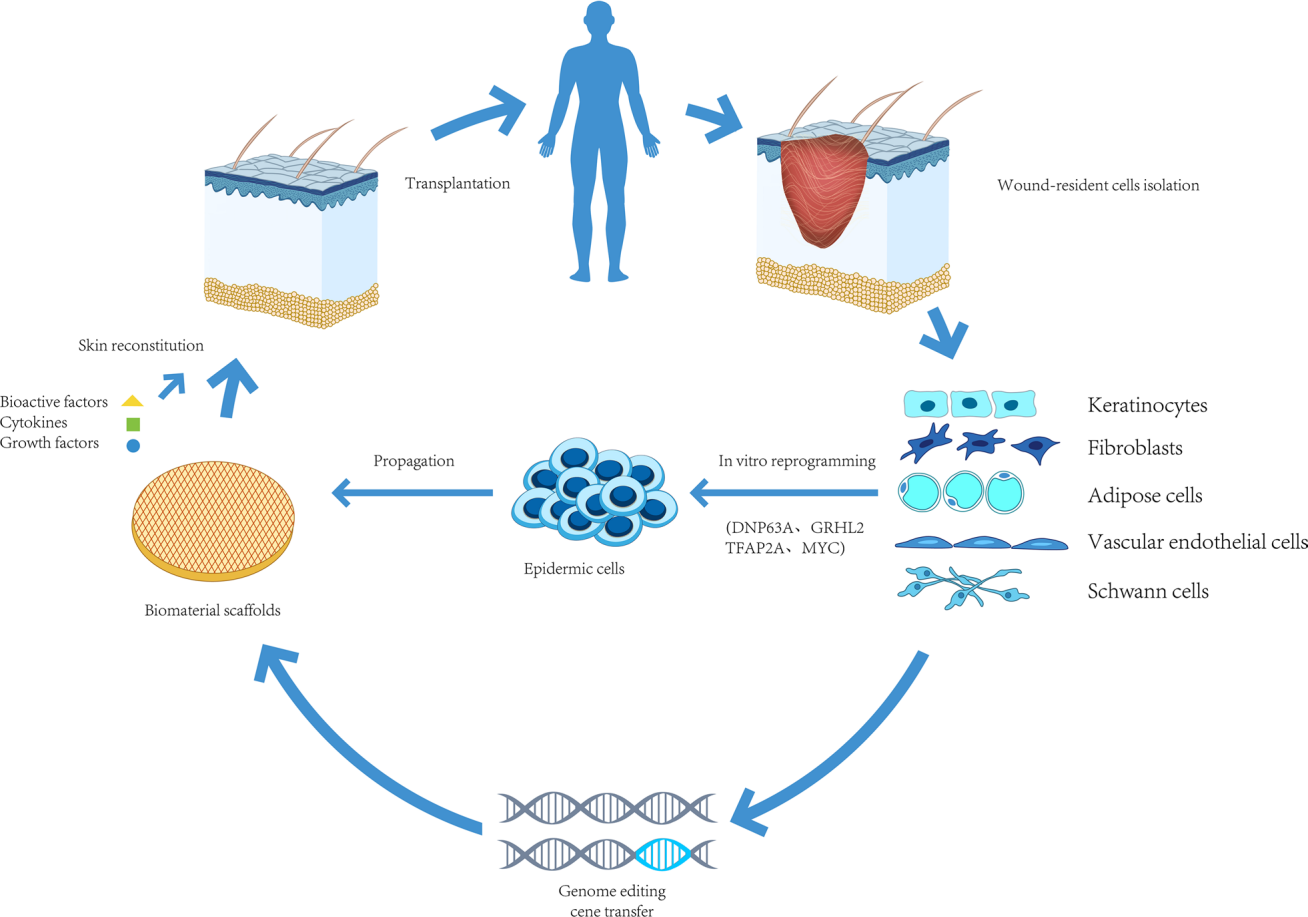
Schematic representation of tissue-engineered skin. Wound resident cells could be obtained from patient wounds. The common wound resident cells include keratinocytes, fibroblasts, vascular endothelial cells, adipose cells, nerve cells, etc. On the one hand, these cells can then be directly reprogrammed into epidermal cells in vitro. On the other hand, wound resident cells can also be genetically edited. The gene that can promote wound healing is introduced into cells to increase the secretion of cell bioactive factors. Finally, these specially treated cells can then be incorporated into a biomaterial scaffold and implanted back to the patient at the damaged tissue site

Skin appendages include hair follicles, sweat glands and sebaceous glands. The recovery of skin sensory function is an important indicator of cutaneous regeneration. All of the skin appendages and nerves play important roles in the physical, chemical and biological functions of normal skin. Regeneration of appendages and nerves requires the use of stem cells, biomaterial scaffolds, bioactive factors and other growth-stimulating factors (Fig. 2). Among these, stem cells can play a crucial role in the regeneration of skin appendages and skin nerves. Stem cells have unique characteristics and roles, including multi-potential capacity, high proliferative potential to differentiate into cells of more than one lineage, self-renewal ability, and participation in tissue regeneration and repair [8]. Embryonic stem cells (ESCs) are capable of giving rise to cell types of all tissue lineages. However, their applications in tissue engineering are constrained by a lack of fundamental understanding and control of their differentiation towards desired specific tissue lineages, as well as ethical debates [9, 10]. In contrast, bone marrow-derived mesenchymal stem cells (BMSCs) are easy to isolate and culture with minimal ethical concerns, expand in vitro, show low levels of immune rejection and low risk of triggering teratoma formation, and can home to injured tissues or organs, including the skin [11–14]. Many clinical trials have demonstrated that BMSCs can leave their niche to migrate to remote tissues and play a critical role in wound repair and tissue regeneration. Therefore, they seem to be an ideal cell source for skin and appendage regeneration [15]. Induced pluripotent stem cells (iPS cells) are reprogrammed by introducing exogenous genes from somatic cells. They are similar to ESCs in function and phenotype and have the ability to expand indefinitely and maintain normal karyotype. In 2006, the University of Tokyo successfully established iPS cells [16] for the first time. In this study, four retroviral vectors carrying OCT 3/4, Sox2, c-myc and Klf4 transcription factors were used to infect fibroblasts and then screened for iPS cells. IPS cells have multi-directional differentiation potential, and can differentiate into all cells derived from 3 germ layers in vivo, making them a good choice for seed cells for disease treatment. IPS cells can differentiate into epithelial cells under specific conditions. Therefore, it is completely possible and feasible to regenerate skin appendages and sensory nerves with iPS cells. Hair follicle stem cells (HFSCs) can be obtained easily and isolated from the skin. HFSCs are located in a specialised niche within the outer root sheath of the hair follicles, known as the bulge [17, 18]. HFSCs can differentiate not only into intact hair follicles but also into sebaceous glands, epidermal keratinocytes and other cells [19, 20]. These options indicate the potential to regenerate skin appendages. Other stem cells, such as human umbilical cord mesenchymal stem cells (hUC-MSCs) derived from Wharton’s jelly, have also been reported to be suitable for skin appendages applications [21].

Biomaterial scaffolds provide seed cells with an environment for adhesion, growth, proliferation and metabolism, which are also important for skin appendages reconstruction and nerve regeneration. A number of biomaterials have been developed for possible skin-replacement therapies. Initially, pure biological materials were extracted and treated to act as scaffolds for cultured cells, and later, various synthetic and semi-synthetic materials were used to either support existing tissues and protect them, or replace them altogether [22]. Biomaterial scaffolds, whether biological, semi-synthetic or synthetic, must meet many requirements, of which the most essential, i are (a) safety, (b) efficacy and (c) convenience [6, 22]. Synthetic biomaterials used for skin-replacement therapies must degrade in a controlled manner in vivo, enhance normal healing of tissues, prevent wound desiccation, and protect the wound from infection. In addition, biomaterials must be easily manufactured, readily available, economical and have a long shelf-life.

Bioactive factors, including various cytokines and growth factors, are also important and play vital roles in tissue reconstruction and regeneration. A variety of bioactive factors have been shown to promote cell proliferation and differentiation, accelerate vascularisation, regulate wound inflammation, and promote appendage regeneration and nerve repair; they also have potential applicability in wound repair and tissue regeneration. Some bioactive factors, such as recombinant bovine basic fibroblast growth factor (rb-bFGF), granulocyte-macrophage colony stimulating factor (GM-CSF) and human granulocyte colony stimulating factor (hG-CSF), have already been developed commercially and gradually adopted in clinical applications [23–25].

Tissue-engineered skin is an inspiring alternative for skin appendages and sensory nerve regeneration. Cells including stem cells, biomolecules, and biomaterials have been widely used to establish a functional vascular and nerve network and induce in situ wound healing, tissue regeneration. However, patients often complain about a loss of skin sensation and cutaneous chronic pain. Restoration of pain, temperature, and touch perceptions should be a major challenge to solve in order to improve patients’ quality of life. Optimisation of existing TES constructs or development of novel TES products, especially those involving skin appendages and sensory nerve regeneration, is a major trend in the field. Herein, we introduce the recent advances and key aspects of different strategies for skin appendages and sensory nerve regeneration, followed by descriptions of current challenges, and future directions.

## Hair follicles

### Structure and functions of hair follicles

Hair follicles reside in the dermal layer of the skin and are made up of hair papillae, hair matrix, root sheath and hair bulges. The papilla is a large structure at the base of the hair follicle, which is composed mainly of connective tissue and a capillary loop. The papilla is surrounded by the hair matrix. The root sheath is composed of an external and internal root sheath. Finally, the bulge is located in the outer root sheath at the insertion point of the arrector pili muscle. It houses several types of stem cells, which supply the entire hair follicle with new cells, and takes part in healing of the injured epidermis [26, 27]. Hair follicles are widely distributed throughout the body, with only a few exceptions, such as the palmoplantar skin, lips and glans penis [28]. Hair follicles are important accessory organs of the skin, with a unique structure and the ability to periodically regenerate. In addition to forming a physical barrier, hair follicles may also exhibit antibacterial ability and inhibit scar formation.

### Signalling pathways and molecules for hair follicle development

To date, at least three main signalling pathways have been confirmed to participate in hair follicle development, i.e. the Wnt, Sonic hedgehog (Shh) and bone morphogenetic protein (BMP) signalling pathways. Activation of the Wnt signalling pathway is required to form hair follicles [29, 30]. Inhibition of Wnt signalling completely abrogates folliculogenesis, and overexpression of Wnt ligand increases the number of regenerated hair follicles [30]. Several Wnts are expressed to mediate maintenance of the hair follicle, including Wnt3a, Wnt5a, Wnt7a and Wnt10b [31, 32]. Wnt3a and Wnt10b are expressed in hair follicle substrate cells. Wnt10b is the earliest and the most prominent molecular signal expressed in the basal plate. Epidermal cells treated with Wnt10b show cell differentiation, to produce the hair shaft and inner root sheath. Abnormal Wnt3a can result in shortening of the hair. Wnt4 can affect the mesenchymal–epithelial interaction, Wnt5a regulates the formation of the dermis, and Wnt7a adjusts the polarity of the cells. The Wnt inhibitor DKK [33] was reported to activate β-catenin [18, 20] and also plays a role in follicle development; Shh signalling is another essential pathway [34]. Gli2 is the key mediator of the Shh responses involved in skin hair follicle development, controlling the transcription of cell cycle regulators to promote proliferation [35]. The third important signalling pathway is the BMP signalling pathway [36]. BMP regulates the growth cycle of hair follicles by regulating the differentiation and proliferation of hair stromal precursor cells during hair follicle morphogenesis [37]. Ectopic expression of BMP4 or specific deletion of the BMP antagonist Noggin, which enhances BMP signalling, results in delayed and more severe hair follicle loss. Overexpression of Noggin induces transition of the hair follicles to the growth phase and disrupts hair shaft differentiation [38]. In addition, when dermal papilla cells cannot receive BMP signals, they lose signature characteristics in vitro and fail to generate hair follicles when engrafted with epithelial stem cells in vivo [39]. However, in another report, BMP signalling was shown to negatively regulate hair bulge stem cells [40], and TG-β signalling counterbalances BMP-mediated repression of HFSC activation [41]. Although many factors have been reported to be involved in hair follicle development, the mechanisms underlying the interactions between cells remain unclear.

### Cells and materials applied in hair follicle regeneration

Hair follicle regeneration can be achieved by applying a number of different types of stem cells [42]. Stem cells with the potential to differentiate into hair follicles are described below.

The implantation of porcine embryonic skin precursors (PESPs) to generate hair follicles and other skin appendages has been reported. Huang et al. [43] first reported that all of the E35–E91 PESPs isolated from Guizhou mini pig embryos exhibited the capacity to grow and generate skin and E56 skin precursors; they can directly instruct skin precursors to generate skin without the risk of teratoma formation. Further studies in immune-competent animals are required before PESP transplantation can be applied in humans.

Recent advances regarding the utilisation of BMSCs have been reported [44, 45]. Green fluorescent protein (GFP)-labelled BMSCs were found in the bulge region of hair follicles, the epidermis, and sebaceous glands [45, 46]. Deng et al. [47] transplanted CM-DiI fluorescently labelled Fik-1+ BMSCs of BALB/c mice (H-2Kd, white) into lethally irradiated C57BL/6 mice (H-2KB, black). Forty days later, the recipient mice grew white hairs in regions that were largely composed of donor-derived H-2Kd cells. Although BMSCs are more accessible and autologous compared to ESCs, adult stem cell properties are contingent on culture conditions and donor factors, and can show loss of proliferation capacity and multipotency [42].

The dermal papilla is widely recognised as the key signalling centre for hair follicle regeneration, which is responsible for maintaining hair growth and controlling the complex hair follicle cycling system [48]. Oliver reported that the implanted dermal papilla regenerated new hair follicles from the outer root sheath [49]. Removal of the lower third or more of the follicles led to permanent cessation of hair growth, indicating the importance of dermal papilla cells [50]. However, the prolonged cultivation of dermal papilla cells has been a major challenge because of the loss of hair follicle-inducing ability and growth activity after several passages. It is possible to maintain hair-inducing ability during long-term cultivation by adding epidermal cell culture conditioned medium. Epidermal growth factor (EGF), fibroblast growth factor-2 (FGF-2), platelet-derived growth factor-A (PDGF-A), hepatocyte growth factor (HGF), insulin-like growth factor-1 (IGF-1) and ascorbic acid-2-phosphate were shown to stimulate follicle growth and promote the formation of a club hair-like structure [51–54].

In recent years, methods of hair follicle regeneration based on tissue engineering have been developed (Table 2). Lee et al. [55] established a simple method to obtain epidermal and dermal cells from neonatal rats, and then prepared high-density cell suspension droplets on culture plates, inoculated them onto the collagen side of Integra dermal substitute, and finally transplanted them to full-thickness skin defects. Eight days after transplantation, hair germ cells formed the hair root base, and a complete hair follicle was formed after 12 days. This hair follicle showed a growth cycle lasting at least 1 year. However, there are many difficulties associated with maintaining the induction ability of human-derived dermal papilla cells under in vitro culture conditions. Most experiments that successfully induced hair follicle regeneration used murine or embryo-derived cells. Human-derived outer sheath keratinocyte and dermal papilla cells can form a tube-like structure when co-cultured in Matrigel, and develop into epidermal cyst-like cell spheres. However, they cannot form intact hair follicles [56]. In another similar study, mouse tissue dermal papilla cells and human keratinocytes were used to inoculate TES formed by collagen glycosaminoglycan matrix. Transplantation into nude mice resulted in the formation of new hair follicles. However, hair follicles did not form when human dermal papilla cells were used instead of murine dermal papilla cells [57]. Qiao et al. [58] reported that dermal papilla cells from the human scalp combined with embryonic mouse epidermis can also induce mature hair follicle formation via skin flap transplantation.

**Table 2:** Methods for regeneration of skin appendages and nerves

	Scaffolding/materials	Cells	Results	References
Hair follicles	Integra dermal substitute (type I Collagen)	Epidermal and dermal cells from neonatal rats	Hairs can be seen as early as 11–15 days postgraft; high reproducibility of hair formation; hair filament shows a normal appearance	[55]
	Matrigel	Human-derived outer sheath keratinocyte and dermal papilla cells	Form a tube-like structure; develop into epidermal cyst-like cell spheres; cannot form intact hair follicles	[56]
	3D-bioprintering (type I collagen gel containing dermal fibroblasts (FBs), microfabricated plastic molds)	Hair papilla cells	Simulate the three-dimensional growth environment; successfully regenerated the skin tissue containing hair follicles Formation of microvascular vessels	[59]
Sebaceous glands	The transplantation of the bioengineered hair follicles	Embryonic skin-derived epithelial and mesenchymal cells	Develop histologically correct hair follicles; sebaceous glands show positive staining with oil red O	[67]
Sweat glands	Scaffolding/materials-free	Heated SGCs co-cultured with BrdU/GFP-labelled MSCs	The MSCs had acquired the sweat gland cell phenotype	[10]
	3D-bioprintering/Matrigel basement membrane matrix	SGCs	Simulate the tissue structure of the sweat gland in vivo	[103]
	EGF-containing gelatine microspheres	SGCs	Develop Sweat gland-like structures	[104]
Skin nerves	Dermal Regeneration Template (collagen–chitosan dermal scaffold)	Schwann cells, skin-derived precursor stem cells, BMSCs, iPS cells	Promote nerve growth, accelerate nerve regeneration	[135–138]

The prerequisite for the application of hair follicle regeneration in clinical treatment is that the cells need to be of ethnic origin. However, most of the hair papilla cells studied for hair follicle regeneration are of mouse origin. Few studies have shown that human hair papilla cells cultured in vitro can regenerate intact hair follicles. The main reason is that human hair papilla cells quickly lose their induction ability in vitro culture conditions. How to maintain the induction ability of dermal papilla in vitro culture is a major problem. The induction function of hair papilla cells to hair follicles and the expression of characteristic genes of dermal papilla are highly dependent on their internal microenvironment. After in vitro culture, the expression of many characteristic genes of hair papilla cells, such as (Akp2, Alx3, Alx4), decreased rapidly, accompanied by a decline of hair follicle induction ability. In summary, the greatest challenge in hair follicle regeneration is how to improve the conditions of in vitro three-dimensional (3D) culture, which in turn would truly simulate the growth environment of hair follicles in vivo, and maintain the biological characteristics of dermal papilla cells and HFSCs to realise reconstruction of mature hair follicles in vitro.

At present, few researches on biological 3D printing to regenerate hair follicles. Abaci et al. [59] simulated the three-dimensional growth environment of human hair papilla cells with a 3D printed mold, successfully regenerated the skin tissue containing hair follicles, and observed the formation of microvascular vessels. There are also studies that observed the regeneration of hair follicles in vitro by mixing mouse fibroblasts and epidermal cells to make a suspension and dropping it on the surface of a 3D printed scaffold [60]. However, there has not been a truly successful study on applying biological ink containing skin cells to biological 3D printing skin and inducing the generation of new human hair follicles. Biological 3D printing for regenerating hair follicles requires a suitable microenvironment for hair follicle growth. This microenvironment is able to maintain the function of hair papilla cells. The microenvironment has an important influence on the proliferation and differentiation of cells and even the entire living organism. Biological 3D printing technology can simulate the microenvironment of hair follicle in vitro and induce hair follicle regeneration. Therefore, biological 3D printed hair follicle regeneration has opened up new ideas for skin tissue engineering technology and provided new solutions, which can greatly promote the development of skin tissue engineering and regenerative medicine.

## Sebaceous glands

### Structure and functions of sebaceous glands

Sebaceous glands are important accessory organs of the skin. Most of the sebaceous glands are located between the hair follicle and the arrector pili muscle, and consist of one or more vesicles as well as a common short duct. The catheter consists of stratified epithelium, most of which opens in the upper part of the hair follicle, although some directly opens on the skin surface. There is a layer of immature cells with abundant organelles and the ability to generate new acinar cells around the acinus through active cell division. Sebaceous glands are distributed over the entire skin surface, except on the palms of the hands and soles of the feet. They show the highest densities on the scalp and face. The main functions of sebaceous glands are to secrete sebum and lubricate the skin. The sebaceous glands also form part of the body’s integumentary system and serve to protect the body against microorganisms. Sebaceous glands secrete acids that form the acid mantle, which is a very slightly acidic film on the surface of the skin that acts as a barrier to bacteria, viruses, and other potential contaminants that may penetrate the skin. Sebaceous glands also have antioxidant effects. Vitamin E, which is transported and secreted by the sebaceous glands, is the main component of the skin’s antioxidant system [61]. The functions of the sebaceous glands are mainly regulated by the endocrine system. Androgens are the primary factor affecting sebum synthesis and secretion, regulating the differentiation and proliferation of the sebaceous glands.

### Signalling pathways and molecules for sebaceous gland development

In contrast to the process of hair follicle morphogenesis, the cellular and molecular mechanisms that control morphogenesis during sebaceous gland organogenesis are still unknown [62]. For many years, sebaceous gland formation has been a technical challenge. Three main pathways and regulatory molecules have been shown to be important for sebaceous gland development, i.e. the Wnt signalling pathway, the Sonic hedgehog (Shh) signalling pathway and the c-Myc signalling pathway [62, 63]. The suppression of Wnt/β-catenin signalling in stem and progenitor cells of mammalian skin can increase sebocyte cell specification and the formation of sebaceous glands. Previous studies demonstrated that mutations in β-catenin, which reduced Wnt activity by directly binding to it and recruiting Smurf2, resulted in sebaceous gland proliferation in mice. In contrast, expression of the Wnt/β-catenin signalling mediator, T cell factor 3 (TCF3), in mouse epidermis suppressed sebocyte transcriptional regulators, resulting in a lack of sebaceous gland formation in vivo [64]. Hedgehog pathway activation leads to marked increases in both the size and number of sebaceous glands [29]. Allen et al. [29] reported that sebocyte fate is governed by the relative levels of stimulatory (hedgehog) and inhibitory (Wnt) signals acting on multipotent progenitors. The myc gene, which encodes the c-Myc protein, is a downstream target of the β-catenin/T cell factor transcription factor. Overexpression of c-Myc lead to increases in both the size and number of sebaceous glands [65]. Although partial sebaceous gland formation has been demonstrated at the molecular and cellular level, further studies are needed to define the precise signalling pathways underlying sebaceous gland formation [29].

### Cells and materials applied in sebaceous gland regeneration

Hair follicle bulge cells with self-renewal capacity and slow periodicity do not only differentiate into the epidermis, but also into skin appendages, including hair follicles, sweat glands, sebaceous glands, etc. Panteleyev et al. [66] examined the skin from 30-day-old mice, and reported that the hair follicle bulge area is surrounded by sebaceous gland cells, and that the degree of differentiation of sebaceous cells is greater farther away from the bulge area. There is indeed a relationship between hair follicles and sebaceous gland regeneration. Asakawa et al. [67] regenerated hair organs by bioengineered hair follicular unit transplantation; sebaceous glands, showing positive staining with oil red O were found to have regenerated in the upper portions of the transplanted bioengineered hair follicles. Oshima et al. [68] directly transplanted the bulge region of murine vibrissal follicles, and sebaceous glands were consistently observed from week 4 after transplantation. These observations further demonstrated that the signals required for sebaceous gland morphogenesis are contained in the upper portion of the follicle. The hair follicle bulge region could be a source of stem cells for the development of hair follicles and sebaceous glands [68, 69].

In addition to HFSCs, other cells were also reported to reproduce sebaceous glands. GFP-positive bone marrow cells were transplanted in a mixture of embryonic mouse skin cells onto murine skin defects, and GFP-positive cells differentiated into sebaceous gland cells within 3 weeks [70]. In another study, porcine mesenchymal stem cells (MSCs) were isolated and engrafted into porcine skin, and the labelled transplanted MSCs were shown to have transdifferentiated into sebaceous duct cells [71].

The bulge area located at the level of insertion of the arrector pili muscle is considered to be a site of pluripotent epithelial stem cells [66]. This hypothesis was supported by the results of transplantation experiments in which isolated keratin 15-positive bulge stem cells were grafted to analyse cell fate plasticity, and it was demonstrated that they were capable of forming all cell linages, including hair follicles and sebaceous glands [19, 66, 68, 72]. However, bulge stem cells are not the only cell source for sebaceous gland renewal. Another hypothesis is that unipotent progenitor cells residing close to the entrance of the gland are dedicated to differentiation into cells of the sebaceous lineage [73, 74]. Both Blimp1+ and Lgr6+ isthmus cells have been shown to self-renew and repopulate the sebaceous glands [73, 75]. Moreover, genetic lineage tracing experiments indicated that cells in the outer layer of the epithelial sheath, which express Sox9, generate hair follicle and sebaceous gland cells, and ablation of Sox9 resulted in the absence of sebaceous glands [76]. Lrig1-positive progenitor cells can also drive sebaceous gland morphogenesis by asymmetric cell fate decision, thereby generating a pool of differentiating sebocytes during morphogenesis of the pilosebaceous unit [62, 74].

The co-localisation of cells positive for the above specific factors with the developing sebaceous glands was not sufficient to confirm that they were sebaceous progenitors [77]. Further studies are needed to fully elucidate the roles of these positive cells in human sebaceous gland formation and maintenance.

## Sweat glands

### Structure and functions of sweat glands

Sweat glands play important roles in regulating body temperature and maintaining fluid balance. Heat dissipation methods, such as conduction, convection and radiation, cease to function in the human body at environmental temperatures above 30 °C, and the secretion of sweat by sweat gland cells (SGCs) becomes the only means of heat dissipation [78]. The sweat glands are divided into eccrine sweat glands and apocrine sweat glands, the former of which are the main glands responsible for regulating body temperature and sweating [79]. Eccrine sweat glands are distributed over almost the whole of the body surface, with the exception of the lips, external ear canal, clitoris, labia minora, glans penis and nail bed [80]. Eccrine sweat glands possess a coiled secretory portion and a long duct that courses through the dermis to the epidermis. They can be activated by either thermal or emotional stimuli, and excrete sweat onto the surface of the skin [46]. Sweat glands do not regularly renew themselves via cell differentiation. The skin of patients with extensive deep burn injuries is repaired by hyperplastic scarring [81] without sweat gland regeneration; it loses perspiration function, which has a serious adverse effect on quality of life. Therefore, the regeneration of sweat glands in the skin is an important topic in the field of tissue engineering and regenerative medicine [82].

### Signalling pathways and molecules for sweat gland development

Multiple genes and signalling pathways have been confirmed to play roles in sweat gland morphogenesis. A variety of cytokines and signal transduction pathways participate in the process of sweat gland regeneration and play important regulatory roles in the process of sweat gland development [83].

The extracellular signal-regulated kinases (ERKs) are widely expressed protein kinases that act as intracellular signalling molecules in the regulation of various physiological processes, such as cell growth, development, division and death [84]. The core of the ERK pathway is the reaction chain composed of three upstream protein kinases (Raf, MEK, ERK). Activated ERK enters the nucleus and activates downstream genes, such as the proto-oncogenes c-Fos, c-Myc, c-Jun, Egr-1, etc., and then regulates the growth and function of cells [85]. Cytokines related to sweat gland development, such as EGF, FGF-10 and HGF, can activate the ERK signalling pathway. EGF activates the ERK signalling pathway and causes a cascade reaction [86]. In one study involving reprogramming of MSCs into sweat gland-like cells, EGF significantly enhanced the efficiency of reprogramming, whereas the ERK pathway blocker, PD98059, partially blocked the reprogramming of MSCs into sweat gland-like cells [44]. These results suggested that EGF is an indispensable factor for sweat gland development and regeneration. The ERK pathway plays an important role in such reprogramming processes [87].

Hypohidrotic ectodermal dysplasia (HED) characterised by developmental defects of sweat glands, hair and teeth, is caused by ectodysplasin-A (EDA) gene mutation, indicating that EDA signals play indispensable roles in the development of sweat glands. EDA-A1 and EDA-A2 are two functional molecules encoded by the EDA gene. The receptors for these molecules are EDAR and XEDAR, respectively. EDA-A1 is directly related to HED and can release functional domains through hydrolysis. The functional domain connects with EDAR to activate a series of downstream signals, such as receptor adapter EDARADD (EDAR-related death domain) and nuclear factor kappa light chain enhancer of activated B cells (NF-κB), thus promoting the occurrence and development of skin appendages [88]. The NF-κB dimer (p50/p65) is a transcription factor, the inactive state of which is a trimer composed of IκB (p50–p60-IκB). Stimulating factors can phosphorylate IκB kinase (IKK) leading to degradation of IκB, and p50/p60 is released from the trimer in the active form. The EDA pathway, mediated by EDA, EDAR and EDARADD, can activate NF-κB transcription factors through the IKK pathway and participates in the development of skin appendages. Activated NF-κB enters the nucleus and promotes the expression of Shh, cyclin D1, DKK 4, fox gene family, keratin 79 and other genes, which play roles in various stages of sweat gland development [89].

In addition, Lei et al. [90] reported that HGF promotes proliferation of human sweat gland epithelial cells, in a process that also involves β-catenin. β-catenin is a key molecule in the Wnt signalling pathway, and plays an important role in cell proliferation and differentiation. When the Wnt signalling pathway is activated by cytokines, the level of free β-keratin in the cytoplasm increases; furthermore, it undergoes translocation to the nucleus to drive the downstream signalling pathway. Sweat gland regeneration is a complex process involving multiple genes and signalling pathways, and further in-depth research is required to determine the mechanism regulating stem cell reprogramming into sweat gland-like cells, which may involve connections between signalling pathways and downstream genes.

### Cells and materials applied in sweat gland regeneration

Patients with large-area burns often suffer from loss of sweat glands, resulting in severe impairment of thermoregulatory function and a significant reduction in quality of life [91]. Therefore, understanding the methodology of the functional repair and reconstruction of sweat glands remains a crucial topic in burns wound repair and regeneration [82]. In theory, the repair and regeneration of sweat glands can be performed using two methods, i.e. through proliferation and differentiation of sweat gland stem cells in situ [92], and by the reconstruction of sweat glands via sweat gland-like stem cell transplantation [10]. However, because of procedural complications, regeneration in situ is difficult to achieve. With the establishment and rapid development of stem cell technology over the past several years, stem cell transplantation has become the first choice for regeneration.

Epidermal stem cells, as the specific stem cells of skin tissues, are key for skin tissue regeneration, wound repair and remodelling [93]. Therefore, epidermal stem cells can be induced to differentiate into SGCs directly, which is one of the most important pathways to sweat gland regeneration. Song et al. [94] reported that miRNA can induce differentiation of human epidermal stem cells into SGCs via a mechanism that may involve post-transcriptional inhibition of the expression of the target gene, p63, by miRNA, thus restricting the proliferation potential of epidermal stem cells and promoting their differentiation into SGCs.

Mesenchymal stem cells have great potential for sweat gland regeneration. Li et al. [95] BMSCs directly or indirectly co-cultured with heat shocked confluence human SGCs can result in converting BMSCs into phenotype of sweat gland-like cells (SGCs) and pERK signals are involved in the course. However, there are certain difficulties in using this approach to regenerate and repair sweat glands. First, the patient’s own bone marrow needs to be extracted, which increases the patient’s mental and physical pain. Secondly, induction in vitro takes a long time, so it is difficult to obtain a large number of sweat gland-like cells derived from stem cells in time. Finally, due to the small sample size in clinical experiments, the long-term clinical practical effect still needs to be observed. Therefore, whether sweat gland-like cells can be directly and effectively applied to escharectomy wounds in patients with large-scale deep burns to realize sweat gland regeneration needs further clinical research. Hao et al. [96] studied the transformation of hUC-MSCs into SGCs and examined the influence of EGF on their transformation and the role of the ERK signalling pathway in the transformation process. The sweat gland-like cells obtained by the method can be used for sweat gland regeneration in the early stage of burn treatment. However, its detailed biological characteristics, safety and whether it can be successfully differentiated into sweat gland tissue after being applied to human body need further research and confirmation. Sheng et al. [10] heated SGCs co-cultured with BrdU/GFP-labelled MSCs at 47 °C for 40 min. After 72 h in culture, GFP tracing and fluorescence staining analyses indicated that the MSCs had acquired the sweat gland cell phenotype. For further analyses in vivo, MSCs co-cultured with SGCs were subcutaneously injected into the paws of nude mice. The results clearly indicated that the transplanted MSCs that had undergone trans-differentiation were involved in the regeneration of sweat glands. In addition to these experiments in animal models, human in vivo testing has also been performed. The co-cultured cell suspension was first covered with a sheet of decellularized allogeneic dermal matrix. Then, granulated autologous skin grafts were laid evenly on top of the sheet to allow epidermal healing with adequate spaces for the exit of the ducts. The regenerated sweat gland-like cells were shown to have acquired the phenotype of SGCs, as well as perspiration function [10]. Although the number of human trials performed to date has been relatively small and the planting area is limited, these observations provided new insights and methods for further exploration of regenerative exocrine sweat gland repair. Another way to obtain the desired sweat glands is the application of MSCs with non-haematopoietic cells to form a heterokaryon, or transfection of MSCs with functional genes [97]. Ectodysplasin A (EDA), which can activate the NF-κB transcription factor through the EDA receptor, has been shown to play a specific role in skin appendages formation [98, 99]. Sweat gland germ cells failed to form in EDA mutant Tabby mice, but were restored by EDA transgene or recombinant EDA [100]. Recently, Cai and colleagues [15] used BM-MSCs transfected with EDA, and the BM-MSC/EDA(+) cells were then transplanted into the injured areas of animal burn injury models. Immunohistochemical analysis and a perspiration test indicated that the combined BM-MSCs improved the regeneration of sweat glands, in terms of both structure and function. The combination of gene therapy and stem cell therapy brings new hope for the functional reconstruction of skin [101].

It has recently become possible to achieve 3D reconstruction of the eccrine gland structure in vitro [102]. Hu et al. [103] used sweat gland tissue digested and separated by collagenase type II to perform 3D culture in vitro, and carried out morphological analysis on the constructed 3D tissue. The 3D-cultured SGCs were inoculated into the tissue structure formed by Matrigel basement membrane matrix to simulate the tissue structure of the sweat gland in vivo. A recent study examined whether sweat glands could be integrated into engineered skin constructs. SGCs were cultured on EGF-containing gelatine microspheres to form a sweat gland cell–microsphere complex. This complex was then transported into the engineered skin construct, in which human keratinocytes were cultured on top of a fibroblast (Fb)-embedded collagen-based matrix, to form an organotypic co-culture model. Sweat gland-like structures developed upon culture of these complexes on engineered skin constructs in vitro [104]. Li et al. [105] cultured SGCs using artificial basement membrane matrix, and reported the formation of tubule-like structures, which are important indicators of SGCs derived from sweat glands and stem cells, and of their biological functions. The above research shows that it is feasible to construct a new generation of tissue engineered skin containing sweat glands. However, how to use stem cells with sweat gland phenotype as seed cells to construct functional tissue engineered skin needs further research and exploration.

Sweat gland regeneration is a complex process involving multiple genes and signaling pathways. And the regeneration of sweat gland requires more in-depth research on the mechanism that regulate stem cells reprogramming into sweat gland-like cells. Further explore the function of sweat gland development-related factors, find key genes involved in sweat gland tissue formation and their regulatory characteristics, and provide new technical methods for the reconstruction of sweat gland function in skin wound repair.

## Skin nerves

### Structure and functions of skin nerves

**Fig. 3:**
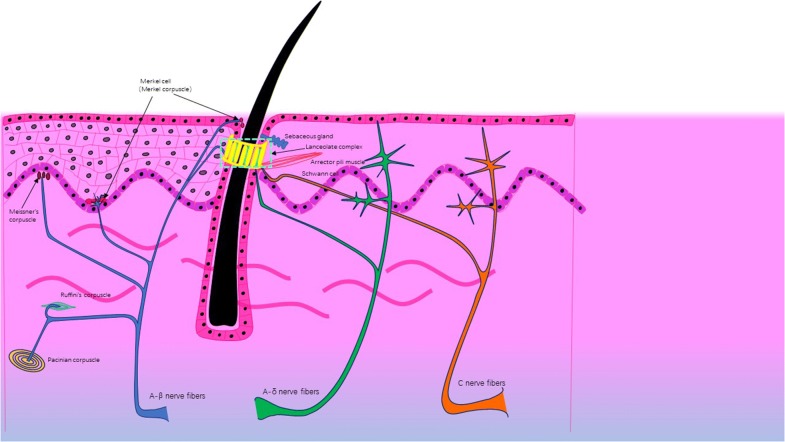
Distribution of skin sensory nerves and receptors

The neural network in the skin is widely distributed and has a complex structure. Cutaneous innervation involves sensory and motor nerves; the former play a major role in a number of skin functions. There are three main types of sensory nerve fibres distributed on the skin, i.e. unmyelinated C group fibres responsible for touch, temperature and pain sensation, A-δ fibres involved in the perception of mechanical stimuli, temperature, and rapid-onset pain, and A-α fibres that respond to light tactile pressure and proprioception [106]. In addition, various sensory nerve endings are widely distributed in the skin, including free nerve endings (FNEs) and specialized nerve endings. FNEs have no complex sensory structures, and they are the most common type of nerve endings in the skin, penetrating the dermis and ending in the stratum granulosum. FNEs infiltrate the middle layers of the dermis and surround hair follicles, and can detect temperature, mechanical stimuli (touch, pressure, stretch) and dangerous stimuli (nociception). Specialized nerve endings include tactile corpuscles, lamellar corpuscles, Meissner’s corpuscles, etc. [107]. Tactile corpuscles are encapsulated unmyelinated nerve endings, which consist of flattened supportive cells surrounded by a connective tissue capsule. Tactile corpuscles are sensitive to shape and texture changes [108]. Lamellar corpuscles, or Pacinian corpuscles, are nerve endings in the skin sensitive to vibration and pressure [109]. Meissner’s corpuscles are encapsulated nerve endings attached to the epidermis in the dermal papillae that detect changes in texture and vibrations [110] (Fig. 3).

The motor nerves of the skin are mainly sympathetic nerves, although there are a few parasympathetic nerves in the face. Although there are few skin motoneurons, they also play an important role in normal skin. The skin motoneurons, which are mostly distributed in the dermis, mainly secrete neurotransmitters, neuromodulators and neuropeptides to regulate important functions, such as sweat gland secretion, vasoconstriction and temperature balance adjustment [111].

### Signalling pathways and molecules for skin nerve development

The skin can produce and secrete neurotrophic factors (NTFs). When the skin is injured, NTF synthesis is increased and their activities are enhanced, thus protecting the damaged neurons and promoting the repair of peripheral nerves [112, 113]. This section will introduce the common signalling pathways and related molecules involved in skin nerve recovery through NTFs. The NTFs are generally thought to activate signalling pathways and effector neurons by binding to receptors, thus providing nutritive support and promoting nerve development [114]. The most common NTFs belong to the neurotrophic (NT) family, including nerve growth factor (NGF), glial cell line-derived neurotrophic factor (GDNF), neurotrophin-3 (NT-3), neurotrophin-4 (NT-4), etc.

The first growth factor to be identified, NGF, was shown to play important roles in the nervous system, angiogenesis and wound healing [115]. NGF can promote peripheral nerve regeneration [116] through two main mechanisms. (i) NGF binds with the axon terminal trkA receptor to form an NGF-receptor complex, which is swallowed by the cell body to form an endosome; this is then transported along the axoplasm towards the nucleus. Next, the endosome activates ERK in the vicinity of the nucleus, and transmits the signal into the nucleus to produce biological effects by regulating the neuronal development, proliferation, differentiation and expression of genes involved in neuronal function. (ii) NGF binds to membrane receptors during transmembrane signal transduction to form dimers and effect autophosphorylation, which activates receptors. The activated receptors induce a cascade of various intracellular proteins and enzymes, in turn triggering signalling reactions. The extracellular signals are then transduced into the nucleus, where they regulate the expression of target genes and produce various structural and functional proteins [117, 118].

Glial cell line-derived neurotrophic factor was first isolated from the conditioned medium of the B49 mouse glial cell line in 1993 [119]. GDNF was originally found to promote the survival of dopaminergic neurons and induce repair of damaged dopaminergic neurons. GDNF is the most biologically active motor NTF identified to date. GDNF can maintain the activity and promote the growth of motor neurons cultured in vitro, and can prevent the atrophy and death of damaged motor neurons [120]. The GDNF receptor expression levels in peripheral nerves and muscles are significantly increased after peripheral nerve injury. Exogenous GDNF can be transported to the cell body through receptor-mediated reverse axonal transport where it plays its neurotrophic role [121]. GDNF promotes nerve growth and repair of nerve injury mainly by interacting with membrane ankyrin receptor GFR to form a GFL/GFR complex, which in turn binds to RET on the surface of the cell membrane to activate the RAS/MAPK and PI3K/AKT signalling pathways [122].

Neurotrophin-3 is a protein growth factor that exerts effects on certain neurons in the peripheral and central nervous systems; it helps to support the survival and differentiation of existing neurons, and encourages the growth and differentiation of new neurons and synapses. NT-3 was the third NTF to be characterised, after NGF and BDNF [123]. Mice born without the ability to produce NT-3 showed loss of proprioceptive neurons, and of subsets of mechanoreceptive sensory neurons [124, 125]. NT-3 promotes neuronal development and survival through members of the signal-mediated tyrosine kinase receptor (Trk) family, where TrkC is the specific high-affinity NT-3 receptor and TrkA and TrkB are low-affinity NT-3 receptors. In addition, NT-3 can also be linked to the multi-functional transmembrane glycoprotein P75 receptor, but its affinity is low [126, 127]. The combination of NT-3 and the Trk receptor can promote axonal cell apoptosis and survival, but the main effect is mediation of apoptosis.

The efficacy of multiple factors in repairing skin nerve injury is superior to that of single factors. As the levels of various NTFs and their receptors change after nerve injury, and where they participate in nerve repair activities in concert, combining the effects of factors in vivo is more concordant with the actual state of the body [128, 129]. The combined use of GDNF and NGF composite collagen tubes to repair sciatic nerve defects in rats has not only shown excellent nerve regeneration, but also confirmed the migration of Schwann cells, which is an important process in successful axon regeneration following the induction of severe nerve defects [130]. Other studies showed that the combined use of NGF, GDNF and CNTF had a better effect on the recovery of sciatic nerve function than a single factor or combinations of two factors, suggesting that nerve function recovery requires supplementation with various NTFs [131]. Tsui-Pierchala et al. [132] reported that the combination of NGF and GDNF promoted phosphorylation of tyrosine kinase, resulting in enhanced gene expression, metabolism and growth. A study of the synergistic mechanism of action of NGF combined with IGF-1 showed that the PI3-K signalling pathway plays an important role in the axon growth induced by NGF and IGF, and this role may be further regulated by the RAS-MAPK pathway [133].

### Cells and materials applied in skin nerve regeneration

Skin nerve repair and regeneration occur in two ways, i.e. via local stem cell proliferation and differentiation, and the extension of healthy axons. Therefore, mobilisation of stem cells to differentiate into neural tissue may promote nerve regeneration. Skin-derived precursor stem cells can be obtained by simple skin tissue biopsy, and have good multidirectional differentiation potential. They can proliferate and differentiate into nerve cells and glial cells in vitro, and can effectively induce regeneration of skin sensory nerves [134, 135]. The precursor stem cells are not only directly involved in neural repair, but are also involved in the reconstruction of epidermal and dermal cells, suggesting that skin-derived precursor stem cells may be useful for the treatment of nerve injury. In addition, adipose-derived mesenchymal stem cells, BMSCs and iPS cells can be induced to differentiate into Schwann cells, thereby promoting nerve regeneration [136–138]. Many stem cells provide numerous possibilities for nerve regeneration in TES, but the risk of immortalised proliferation due to totipotency or pluripotency of stem cells cannot be ignored [134]. Therefore, a great deal of research will be required to determine how to precisely direct the mature differentiation of stem cells by altering the microenvironment of TES.

Schwann cells are known to play supporting roles in nerve regeneration [139]. When the nerve is damaged, Schwann cells can guide regeneration by forming a tunnel that leads towards the target neurons. The stump of the damaged axon is able to sprout, and these sprouts can grow through the Schwann cell ‘tunnel’ at a rate of approximately 1 mm/day under good conditions. Schwann cells, the main component of the myelin sheath in nerve fibres, were added to collagen–chitosan dermal scaffold, and the results showed that the number of cutaneous neurites in this dermal scaffold was significantly increased [140]. The roles of Schwann cells in promoting nerve regeneration include: (1) proliferation and aggregation to form a tubular structure similar to a ‘nerve conduit’, which induces the directional growth of nerve fibres; (2) synthesising and secreting a large number of laminins (LN), which promote nerve growth and can significantly accelerate the regeneration of class A nerve fibres to accelerate nerve regeneration [141]; (3) secretion of NGF and other NTFs to promote axon regeneration by up-regulating the expression of neuronal cell adhesion molecules [142]. Hence, Schwann cells, as a key cellular component in skin sensory nerve regeneration, are massively expanded and added to the dermis scaffold in vitro, which constitutes a good method for the regeneration of TES sensory nerves.

Following a deep skin defect caused by severe burns, large numbers of Fbs proliferate and accumulate around the wound and nerve endings. Although Fbs are vital for wound healing and tissue repair, they are not conducive to the regeneration of blood vessels and nerves [143]. Therefore, the regeneration of nerve tissue must effectively inhibit wound contraction, especially the aggregation of myofibroblasts (MFbs) around nerve endings [144]. Dermal regeneration template (DRT), as the basic structure of TES, provides strong support for sensory nerve regeneration. DRTs that can effectively promote sensory nerve regeneration must fulfil several requirements: (i) inhibition of the proliferation and accumulation of wound Fbs, especially the proliferation of MFbs around nerve endings; and (ii) appropriate pore size and biodegradation rate, and good biochemical characteristics [143]. Therefore, in theory, a dermal scaffold that inhibits wound contraction and Fb aggregation, and has an appropriate pore size, could promote the regeneration of sensory nerves in TES. However, long-term neurosensory function after conventional dermal scaffold transplantation is poor, which may be related to impaired nerve signal transmission, the specific mechanism of which is still unclear [145].

During the process of TES-induced neo-tissue formation, nerve regeneration is highly dependent on newly formed blood vessels. With lack of blood supply, nerve regeneration is almost completely inhibited. While the vascular embryo is growing, a large number of new nerve fibres surrounding the blood vessels gradually regenerate along with the extension of blood vessels, eventually reaching the epithelial layer to form FNEs or connect with receptors [146]. There are two main mechanisms by which blood vessels likely promote nerve regeneration. First, neovascularisation not only provides nutrition for nerve cells, but also delivers NTFs through the circulation. Second, new blood vessels can also inhibit fibrosis and promote nerve regeneration. Meanwhile, the regenerated nerve can secrete neuropeptides or NTFs, promote the survival, growth, migration and proliferation of endothelial cells, and promote angiogenesis by regulating the expression of angiogenic activity factor. Hence, vascularisation and nerve regeneration are two complementary processes—vascular regeneration creates the conditions for nerve regeneration, and nerve regeneration also provides strong support for the growth of blood vessels.

## Perspectives and challenges

The regeneration of skin appendages is still one of the greatest challenges in the fields of tissue engineering and regenerative medicine. Numerous approaches have been developed over the past several decades to overcome those problems, and some encouraging results have been reported. However, further research is needed to better understand and promote the regeneration of skin appendages. The most important aspect is optimising the design, i.e. skin construction and functional positioning, of TES. The construction of scaffolds is often considered to be a key factor in TES, as scaffolds are not only used as DRTs to induce tissue regeneration, but also as carriers for inoculation of seeding cells and structures modified by growth factors. In the field of biological materials, many materials have been shown to have potential as TES scaffolds, but few TES products have been developed in the laboratory for clinical applications. In addition, as cell components and growth factors are introduced into the scaffold to create active tissue, the interactions between cells, matrix material and growth factors become more complex. Therefore, there is a great deal of research on optimising biological materials and structures. Appendage regeneration, rapid vascularisation and nerve repair of the scaffold have also become important indices of the effectiveness of scaffold construction [147]. The directional growth of stem cells results in differentiation and biosafety issues. Stem cells have attracted a great deal of attention as an important source of tissue-engineered seed cells. Recent studies indicated that stem cells can play an important role in promoting appendage regeneration, wound healing and tissue reconstruction [148–150]. Stem cells have a great deal of potential in the field of TES, but the specific mechanisms by which stem cells participate in wound healing are not clear, and it is still difficult to avoid mutation and tumorigenesis in culture. In addition, ethical concerns about stem cells remain. Finally, the application and regulation of several factors is required. Although many studies have demonstrated the good prospects of TES combined with growth factors, commercial development has been relatively slow. There are a number of possible reasons for this: (i) TES contains multiple components that must undergo rigorous testing; (ii) growth factors are expensive and easy to deactivate, and have stringent storage, transportation and production requirements; (iii) the method of introducing growth factors into tissue engineering scaffolds involves a variety of chemical reagents, and their safety, effectiveness and stability raise potential biosafety concerns; and (iv) the application and regulation of multiple growth factors remains a difficult challenge.

Nerve regeneration is a challenging but intriguing topic, which also requires more research attention. Firstly, nerve regeneration is not equivalent to normal functional recovery. Most reports published to date have generally regarded the number of nerves regenerated as an “observation index”, and seldom considered the recovery of sensory function after nerve regeneration (i.e. the quality of sensory nerve regeneration). Therefore, future studies should examine how to promote neural integrity and regeneration. Secondly, in cases of severe skin injury, such as deep burns, the receptors of various nerve fibres are severely damaged. In particular, the receptors of A-β nerve fibres located deep within the skin show very poor regeneration after being completely destroyed. An effective method could promote the growth of nerve fibres into new tissues, but most of the tactile functions cannot be recovered due to the lack of receptors, while the retention of peripheral receptors largely depends on the depth of the skin defect. Although some haptics can be regenerated by reconstructing the follicular structure, the effectiveness of this approach remains to be clinically tested. Further research is required regarding the reconstruction of receptors to achieve the ultimate goal of restoring sensory function of the skin.

## Conclusions

The ultimate goal of TES is the development of a TES equivalent that combines living cells, biomaterials and bioactive factors to mimic all of the functions of human skin. Currently, although many mature TES products are commercially available, most of these products are only structurally similar to human skin, and provide only barrier function. Due to their lack of skin appendages and nerves, these tissues do not have the complete function of the intact skin and thus do not represent true regeneration thereof. The rapidly advancing progress of research on cells and materials brings the goal of achieving advanced TES containing whole skin appendages, blood vessels and nerves closer to fruition. Further steps from bench-to-bed of the research results can be anticipated in the forthcoming decades.

## Data Availability

All data or related information supporting the conclusions of the review is included in the article.

## Abbreviations

TES: Tissue-engineered skin
ESCs: Embryonic stem cells
BMSCs: Bone marrow-derived mesenchymal stem cells
iPS cells: Induced pluripotent stem cells
HFSCs: Hair follicle stem cells
hUC-MSCs: Human umbilical cord mesenchymal stem cells
rb-bFGF: Recombinant bovine basic fibroblast growth factor
GM-CSF: Granulocyte-macrophage colony stimulating factor
hG-CSF: Human granulocyte colony stimulating factor
Shh: Sonic hedgehog
BMP: Bone morphogenetic protein
PESPs: Porcine embryonic skin precursors
GFP: Green fluorescent protein
EGF: Epidermal growth factor
FGF-2: Fibroblast growth factor 2
PDGF-A: Platelet-derived growth factor-A
HGF: Hepatocyte growth factor
IGF-1: Insulin-like growth factor-1
HGF: Hepatocyte growth factor
TCF3: T cell factor 3
3D: Three-dimensional
MSCs: Mesenchymal stem cells
ERKs: Extracellular signal-regulated kinases
HED: Hypohidrotic ectodermal dysplasia
EDA: Ectodysplasin-A
EDAR: Ectodysplasin-A receptor
EDARADD: EDAR-related death domain
IKK: IκB kinase
SGCs: Sweat gland cells
miRNA: microRNAs
FNEs: Free nerve endings
NTFs: Neurotrophic factors
NT: Neurotrophic
NGF: Nerve growth factor
GDNF: Glial cell line-derived neurotrophic factor
NT-3: Neurotrophin-3
NT-4: Neurotrophin-4
iPSCs: Induced pluripotent stem cells
LN: Laminins
Fbs: Fibroblasts
MFbs: Myofibroblasts
DRT: Dermal regeneration template
